# Systemic loss of Sarm1 protects Schwann cells from chemotoxicity by delaying axon degeneration

**DOI:** 10.1038/s42003-020-0776-9

**Published:** 2020-01-30

**Authors:** Weili Tian, Tim Czopka, Hernán López-Schier

**Affiliations:** 1 Sensory Biology & Organogenesis, Helmholtz Zentrum Munich, Munich, Germany; 2 Institute of Neuronal Cell Biology, Technical University of Munich, Munich, Germany

**Keywords:** Genetics, Neuroscience, Neurology

## Abstract

Protecting the nervous system from chronic effects of physical and chemical stress is a pressing clinical challenge. The obligate pro-degenerative protein Sarm1 is essential for Wallerian axon degeneration. Thus, blocking Sarm1 function is emerging as a promising neuroprotective strategy with therapeutic relevance. Yet, the conditions that will most benefit from inhibiting Sarm1 remain undefined. Here we combine genome engineering, pharmacology and high-resolution intravital videmicroscopy in zebrafish to show that genetic elimination of Sarm1 increases Schwann-cell resistance to toxicity by diverse chemotherapeutic agents after axonal injury. Synthetic degradation of Sarm1-deficient axons reversed this effect, suggesting that glioprotection is a non-autonomous effect of delayed axon degeneration. Moreover, loss of Sarm1 does not affect macrophage recruitment to nerve-wound microenvironment, injury resolution, or neural-circuit repair. These findings anticipate that interventions aimed at inhibiting Sarm1 can counter heightened glial vulnerability to chemical stressors and may be an effective strategy to reduce chronic consequences of neurotrauma.

## Abstract (web summary)

Tian et al. showed that systemic elimination of Sarm1 in zebrafish increases Schwann-cell resistance to chemotherapeutics and protects axons from Wallerian degeneration. They use genetics, pharmacology, and high resolution intravital videomicroscopy to study Sarm1 in vivo.

## Introduction

The peripheral nerves that communicate skin, muscle, and sensory organs with the brain must maintain functionality throughout life despite frequent stress and trauma^1–6^. Loss of integrity of peripheral neurons and associated cells, including glia, is a common occurrence in severe neurological dysfunctions that include weakness, pain, and loss of sensation^7^. Glial loss leads to nerve demyelination, defasciculation, and neuronal death. Nerve injury triggers axon fragmentation and degeneration. In turn, this induces the dedifferentiation of associated glial cells, which enhances glial role in nerve repair. However, dedifferentiation also makes glia vulnerable to degeneration after protracted denervation^8–12^. Damaged axons undergo Wallerian degeneration^13^, which is triggered by the evolutionary ancient pro-degenerative protein Sarm1 (Sterile Alpha and TIR Motif containing 1)^14–17^. Sarm1 is a modular protein that contains two sterile-alpha motifs (SAM), one mitochondrial association (MT) and one TIR domain^14^. Nerve injury activates Sarm1 by TIR dimerization, which is sufficient to induce axonal degeneration^18,19^. Mechanistically, Sarm1 activation results in the loss of nicotinamide adenine dinucleotide (NAD+) in damaged axons^20–22^. The TIR domain acts as a NADase^23^, explaining why over-expression of the NAD+-synthesizing enzyme NMNAT1 inhibits the degradation of severed axons by sustaining high levels of NAD+ downstream of Sarm1 (ref. ^24^). Moreover, the loss of Sarm1 blocks the degeneration of injured axons, and forced activation of Sarm1 induces axon destruction in the absence of injury. Therefore, Sarm1 is a hierarchical regulator of a signaling pathway that is necessary and sufficient for axonal degradation^22^. Upon peripheral-nerve injury, glial Schwann cells acquire a specialized function that promotes the clearance of axon fragments ahead of axonal re-growth from the proximal stump^25^. Expeditious axonal regeneration is important because sustained Schwann-cell denervation leads to protracted loss of glial terminal phenotype and eventual death. In turn, glial loss impairs regenerating nerve myelination and circuit repair, transforming acute neuropathies into irreversible chronic neurological dysfunction. Therefore, inhibiting or delaying axon destruction has been hypothesized as an effective strategy to counter heightened Schwann-cell vulnerability to additional stressors that include metabolic imbalance and drugs^26,27^. As a discrete hierarchical factor that is essential for axon degeneration, Sarm1 is an ideal target for pharmacological interventions^18^. This idea has sparked intense efforts to identify specific molecular inhibitors of axon degeneration for clinical applications^28,29^. However, the conditions that would most benefit from inhibiting Sarm1 systemically are yet to be defined^30,31^. Here we address the above issue using the powerful genetics of zebrafish, a small vertebrate whose nervous system is anatomically simple but functionally similar to that of mammals^32–34^. Crucially, the zebrafish larva is ideal to study Schwann-cell biology in the natural context of the behaving animal^35–42^. By characterizing loss-of-function mutations in Sarm1, we provide novel insights into the cellular basis of axon–glia interactions, and provide data that encourage the quest to identify and develop Sarm1 inhibitors for clinical applications.

## Results

### Identification and mutagenesis of Sarm1 in zebrafish

**Fig. 1:**
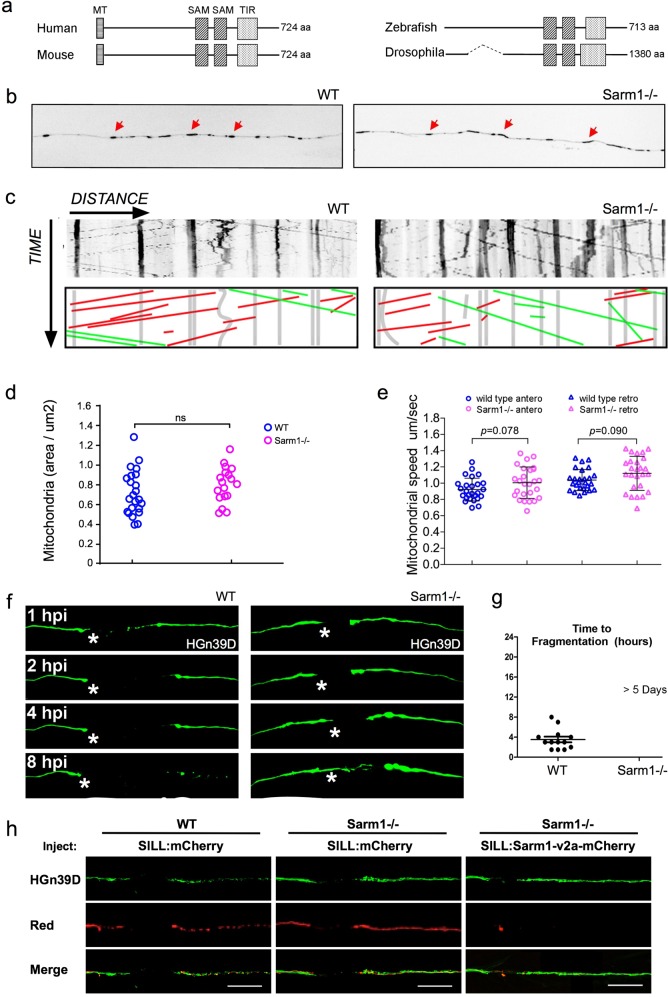
Functional conservation of Sarm1 in zebrafish. **a** Structure Sarm1 indicating alignment of the Sarm1 functional domains from different species (not at scale). **b** Confocal image of axonal mitochondria marked with mito-mCherry in wild type and Sarm1−/−. Red arrows point to prominent mitochondrial groups in axons. **c** Upper panels, kymographs from videomicroscopic recording of axonal mitochondria in wild type (H) (left panel) and Sarm1−/− (I) (right panel). Lower panels show color-coded traces of moving mitochondria in anterograde (green) and retrograde (red) directions, taken from the kymographs shown in the upper panels. **d** Density of mitochondria in 5 dpf wild type and Sarm1−/−, error bar = SEM. n.s. = not significant, *p* value from Student’s *t*-test, *n* = 25 (WT), *n* = 19 (Sarm1−/−). **e** Mobility of the mitochondria in 5 dpf wild type and Sarm1−/−. Circles show the anterograde and triangles the retrograde movement of the mitochondria. *p* value from one-way ANOVA, wild type *n* = 26, Sarm1−/− *n* = 26. **f** Time-lapse images of axonal degeneration of GFP-labeled lateralis sensory neuron in wild type (left) and Sarm1−/− larvae (right). hpi = hour post-injury, scale bar = 50 μm, white asterisk indicates the regrowing axons from the proximal stump. **g** Quantification of the time from axon transection to fragmentation in wild type (*n* = 13) and Sarm1−/− (*n* = 13). **h** shows a recue experiment in which the expression of a functional Sarm1 in sensory neurons of Sarm1-mutant fish (right-hand side panels) suffices to degrade severed axons, similarly to wild-type fish (left-hand side panels). Middle panels show non-degradable Sarm1-deficient severed axons.

The amino-acid sequence of Sarm1 is well conserved across species^15,43^. To identify Sarm1 orthologs in zebrafish, we scanned publicly accessible genomic data (*Danio rerio* reference genome assembly version GRCz11) by a BLAST search using the TIR domain, which is present in all known Sarm1 proteins^15,44^. This exploration yielded a single candidate locus in chromosome 15. No other part of the zebrafish genome appears to harbor Sarm1 paralogs. The genomic structure of the putative zebrafish Sarm1 reminisces that of other species, containing 8 exons that code for a protein of 713 amino acids, with the typical N-terminal auto-inhibitory domain, 2 central SAM multimerization domains, and a C-terminal TIR degeneration domain (Fig. 1a). Similar to *Drosophilla melanogaster*, however, *D. rerio* Sarm1 lacks an obvious mitochondria-targeting sequence (MT). To test whether the identified gene produces a protein with the expected functional role, we used CRISPR/Cas9-mediated genome modification to generate loss-of-function mutations in Sarm1. By targeting exon 1, we obtained germ-line transmission of two alleles: *sarm1*^*hzm13*^ and *sarm1*^*hzm14*^ (Supplementary Fig. a, b). The hzm13 allele introduces an 11-base deletion and T/C mutation, resulting in a frameshift and premature stop codon. hzm14 is a 7-base deletion and AG/GA mutation that also generates a frameshift and premature stop codon. Analysis of protein extracts from wild-type embryos by western blot using an antibody to Sarm1 revealed a single band of approximately 80 kDa, which agrees with the expected size of the full-length protein (Supplementary Fig. 1c). This band was absent in protein extracts from homozygous *sarm1*^*hzm13*^ zebrafish embryos. Of note, because this antibody recognizes an epitope in the C-terminus of Sarm1, it does not allow to discriminate between the expression of a truncated protein lacking all the domains with known function, and the complete absence of Sarm1 induced by nonsense-mediated mRNA decay. Homozygous *sarm1*^*hzm13*^ mutants display no overt anatomical defects (Supplementary Fig. 1d, e), are viable, and develop into fertile adults. Furthermore, a simple assay for sensorimotor function that consists of eliciting the escape response after tactile stimuli showed that the displacement distance and the average acceleration were no different between wild type and Sarm1 mutants (Supplementary Fig. 1f, g)^45^.

### Functional conservation of Sarm1 in axon degeneration

Although Sarm1 has already been extensively studied in neurons of *Drosophila* and mice, we deemed necessary to assess the effects of systemic loss of Sarm1 on neuronal and non-neuronal cells in larval zebrafish. This is because no single study has addressed Sarm1 function holistically, in a single organism, by intravital high-resolution microscopy. To this end, we used several parameters of nervous-system structure and function by combining *sarm1*^*hzm13*^ with transgenes expressing fluorescent markers in the sensory neurons of the mechanosensory lateral line, as well as in their associated Schwann cells^46,47^. The lateral-line system is ideal for such in vivo studies under normal and altered conditions^48–51^. It combines the organization of a typical vertebrate sensory system with the amenability for controlled experimental interventions that include microsurgery, pharmacology, and optogenetics^41,42,52,53^. We found that loss of Sarm1 does not affect the development and structure of the lateral-line sensory pathway (Supplementary Fig. 1h–n). For a more detailed analysis, we fluorescently marked mitochondria in sensory neurons by expressing the mitochondria-targeting sequence of the cytochrome-*C* oxidase subunit 8A fused to mCherry^54^. We chose to study mitochondria because they are dynamic organelles that distribute throughout the neuron by active transport mediated by molecular motors that move along microtubule tracks. In axons, microtubules are polarized such that their plus ends are directed toward the axon terminals, in turn orienting the movement of distinct molecular motors in the antero- and retrograde directions^55^. Intra-axonal movement direction reflects mitochondrial fitness because stressed organelles are biased in the retrograde direction^56,57^. Therefore, axonal mitochondria represent an optimal proxy for neuronal polarization, intracellular dynamics, and overall health. We found a qualitatively similar number, density, and distribution of mitochondria in the peripheral axons of wild type and Sarm1-mutant animals. Kymographic analysis revealed a majority of static large mitochondrial groups, and some smaller fragments moving substantial distances at constant velocity in the anterograde and retrograde directions (Fig. 1b, c). Importantly, quantifications showed no significant differences in the number and spatial distribution of axonal mitochondria (Fig. 1d), or movement velocity and direction between wild-type and Sarm1-mutant specimens (Fig. 1e).

Next, we addressed functional conservation of Sarm1 in zebrafish using a previously established bioassay of neurotrauma in vivo, which employs laser-mediated severing of individualized axons using single-neuron fluorescent-protein expression and high-resolution microscopy^41,58^. To precisely cut axons, we focused an ultraviolet laser beam to a discrete region of the peripheral nerve. Upon severing, the distal-axon segment quickly degenerated in wild-type specimens, whereas the proximal segment that stays associated with the neuronal perikaryon remained viable (Fig. 1f). Kinetic analysis shows that Sarm1-deficient severed axons remained stable for over 5 days, whereas severed wild-type axons degraded within 8 h (Fig. 1g). The degeneration-resistant phenotype of Sarm1 mutants was eliminated by the transgenic introduction of a fluorescent-tagged full-length Sarm1 in mutant neurons (Fig. 1h), demonstrating functional conservation of zebrafish Sarm1.

### Loss of Sarm1 does not affect focal damage resolution

**Fig. 2:**
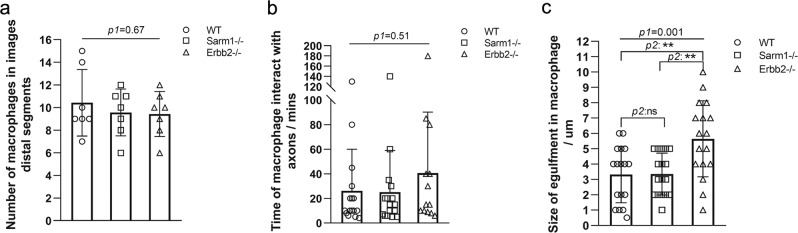
Sarm1 deficiency does not affect damage resolution. **a** Quantification of the number of macrophages recruited to the injury site and adjacent axon segments. **b** Quantification of the time macrophages interact with axon segments. **c** Quantification of the size of debris within macrophages. One-way ANOVA was conducted firstly, then *p* values for *T*-test in between two individual group.

Physical injury often results in inflammatory responses that recruit immune cells to the wound. After nerve injury, injury-mediated production of reactive oxygen species (ROS) as well as chemoattractants produced by Schwann cells recruits inflammatory cells, including macrophages. Studies ex vivo using mammalian cultured neurons showed that Sarm1 acts downstream of mitochondrial ROS generation. Therefore, we decided to test in vivo if the loss of Sarm1 or the absence of Schwann cells would affect macrophage recruitment to the site of injury. To this end, we generated a transgenic line expressing membrane-targeted EGFP under the control of the macrophage-specific promoter Mfap4. We combined this line with Tg[SILL:mCherry] and mutations in Sarm1 or Erbb2. Loss of Erbb2 in zebrafish impairs Schwann-cell migration along lateral-line axons and leads to nerve unmyelination and defasciculation^53,59^. We injured nerves with a laser and assessed macrophage behavior at high resolution. We found that the onset of recruitment and the number of macrophages at the wound did not differ between wild-type specimens and Sarm1 or Erbb2 mutants (Fig. 2a and Supplementary Movies 1–3). Macrophages arrived from various locations and moved in a qualitatively indistinguishable manner along the proximal and distal part of the axons in wild type and mutant specimens. Quantitative imaging showed that the retention time of macrophages at the proximal side of the wound was unaltered in Sarm1 or Erbb2 mutants, although on average retention was marginally lengthened in the absence of Schwann cells (Fig. 2b). In wild type, Sarm1, and Erbb2 mutants, macrophages engulfed debris locally at the injury site, and did not appear to be involved in the degradation of the distal part of the severed axons (Supplementary Movies 1–3). We found, however, a significant increase in the size of the engulfed debris by macrophages in Erbb2-mutant animals (Fig. 2c). These results reveal that the loss of Sarm1 does not affect focal damage resolution by macrophages. Also, importantly, they indicate that macrophages are recruited to the wound independently of the Schwann cells. We did notice discrete and highly mobile axon fragments that were not associated with EGFP(+) cells in wild type and Erbb2-mutant animals, but not in Sarm1-deficient fish.

### Synthetic degradation of severed Sarm1-deficient axons

**Fig. 3:**
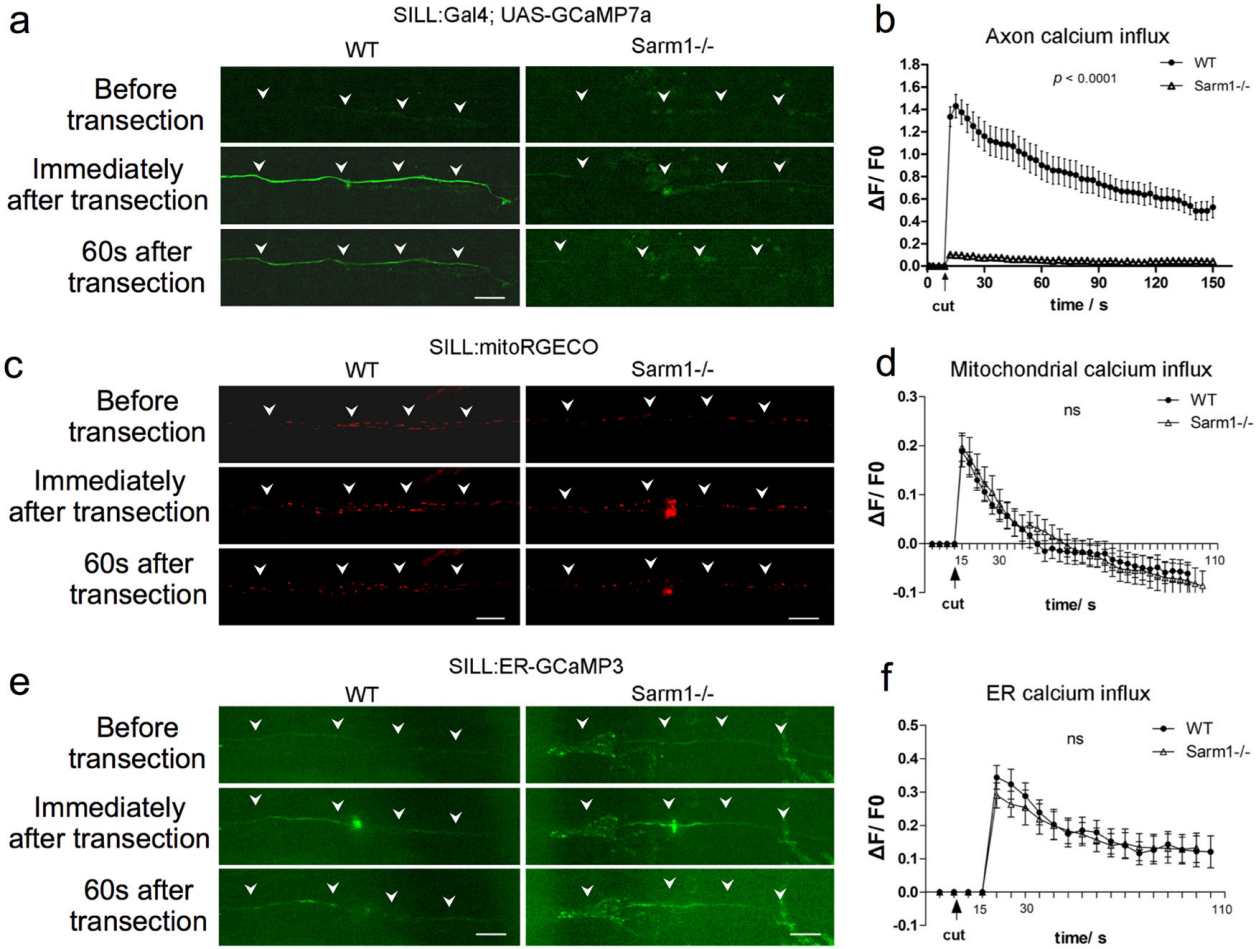
Loss of Sarm1 attenuates Ca^2+^ influx to the axoplasm of severed axons, but not Ca^2+^ uptake in mitochondria or the ER. **a** Confocal image of a single lateralis sensory axon expressing the green-fluorescent calcium sensor GCaMP7a in wild type (left column) and Sarm1−/− fish (right column). Rows show that the same samples before laser-mediated transection (top), immediately after transection (middle), and 60 s after transection (bottom). In **a**, **c**, and **e**, white arrowheads indicate the position of the axon, specifically when signal-to-background is low. Scale bar 20 μm. **b** shows quantification of the first wave of axoplasmic calcium. Data are shown as mean ± SEM; *p* from one-way ANOVA, wild type *n* = 16, Sarm1−/− 16. **c** shows a confocal image of lateralis sensory axons expressing the red-fluorescent calcium sensor RGECO in wild type (left column) and Sarm1−/− fish (right column). Rows show that same samples before laser-mediated transection (top), immediately after transection (middle), and 60 s after transection (bottom). **d** Quantification mitochondrial calcium influx shows the strong and nearly identical elevation and decay in wild type and Sarm1−/− immediately after the cuts. Data are shown as mean ± SEM; *p* from one-way ANOVA, wild type *n* = 16, Sarm1−/− 16. **e** shows a confocal image of lateralis sensory axons expressing the green-fluorescent calcium sensor CCaMP3 targeted to the endoplasmic reticulum (ER) in wild type (left column) and Sarm1−/− fish (right column). Rows show that same samples before laser-mediated transection (top), immediately after transection (middle), and 60 s after transection (bottom). **f** Quantification ER calcium influx shows strong and statistically equal elevation and decay in wild type and Sarm1−/− after the cuts. Data are shown as mean ± SEM; *p* from one-way ANOVA, wild type *n* = 16, Sarm1−/− 16.

Calcium (Ca^2+^) regulation in neurons is critical for homeostasis because sustained elevation of cytosolic calcium leads to axonal and neuronal degeneration. Often, this occurs because mitochondria release apoptosis-inducing factors and proteases in a calcium-dependent manner^60^. Studies of mammalian neurons in vitro and of zebrafish have shown that neuronal damage triggers two waves of elevation of axoplasmic calcium (Ca^2+^)^21,52^. The first occurs nearly immediately after injury and decays rapidly, whereas the second has slower onset and decay. Loss of Sarm1 prevents Ca^2+^ elevation in severed axons^21^. We sought to further test the functional conservation of Sarm1 in zebrafish in vivo by monitoring Ca^2+^ dynamics in lateralis sensory axons at high resolution before and after injury. We measured Ca^2+^ dynamics in toto at high resolution before and after axon injury using the genetically encoded ultrasensitive Ca^2+^ sensor GCaMP7a to find that intact wild type and Sarm1-mutant axons show undetectable levels of axoplasmic fluorescence above background (Fig. 3a). Upon severing, fluorescent signal in wild-type axons distal segments increased immediately and subsequently decayed with a near constant slope, whereas fluorescence remained nearly undetectable in Sarm1-deficient distal-axon segments (Fig. 3b). Next, we examined the Ca^2+^ levels in mitochondria and the endoplasmic reticulum (ER) using genetically encoded vital sensors, respectively, Mito-RGECO and ER-GCaMP3 (Fig. 3c–f). We selected these organelles because Ca^2+^ release from the axonal ER activates the mitochondrial permeability transition pore to trigger axonal degeneration^61,62^. We found that both mitochondrial and reticular Ca^2+^ levels increased equally after axon severing in severed wild type and Sarm1−/− axons. These results reveal that loss of Sarm1 attenuates calcium influx to the axoplasm, but not Ca^2+^ uptake in mitochondria or the ER.

**Fig. 4:**
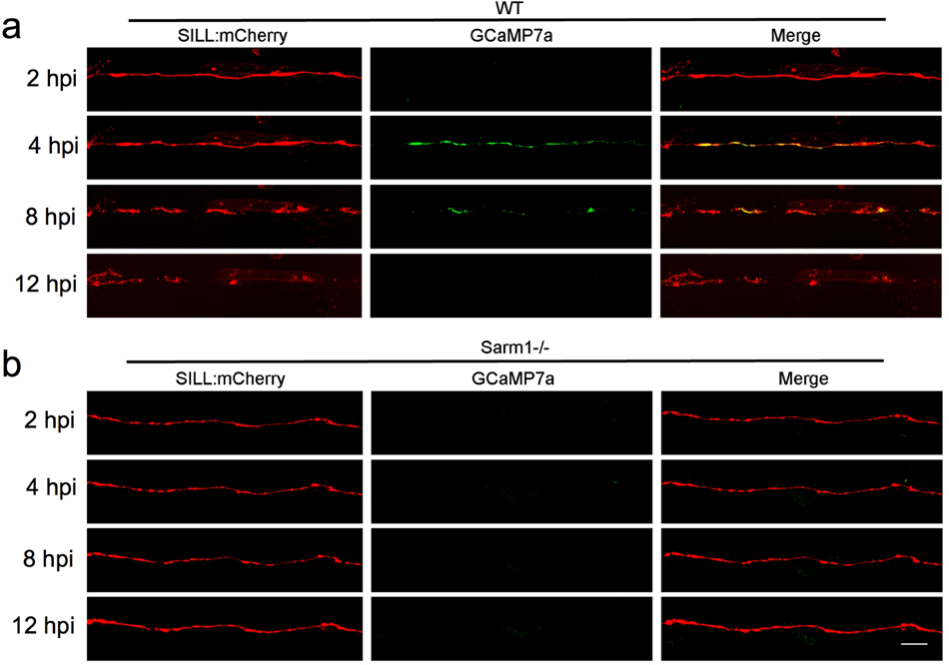
Sarm1 is necessary for late onset axoplasmic Ca^2+^ influx in severed axons. **a** Confocal image of a lateralis sensory axons expressing the mCherry (red) and green-fluorescent calcium sensor GCaMP7a (green) in wild-type fish. Rows show same axons 2, 4, 8, and 12 h after transection (hours post-injury = hpi). **b** Confocal image of lateralis sensory axons expressing the mCherry (red) and green-fluorescent calcium sensor GCaMP7a (green) in Sarm1−/− fish. Scale bar 20 μm.

Because the second Ca^2+^ wave is responsible for the activation of the serine–threonine protease Calpain, which in turn facilitates axonal fragmentation by cleaving microtubules and neurofilaments^63^, we decided to monitor Ca^2+^ levels in lateralis sensory axons 2, 4, 8, and 12 h post-injury (hpi). In severed wild-type axons, the second wave of axoplasmic Ca^2+^ starts 4 hpi in coincidence with axon fragmentation, and remains elevated in axonal debris up until 8 hpi (Fig. 4a). Note that the temporal resolution of these images does not allow the resolution of degeneration before axonal regeneration. By contrast, the second wave of axoplasmic Ca^2+^ does not occur in Sarm1-mutant axons (Fig. 4b). Thus, we hypothesized that forcing a sustained elevation of axoplasmic Ca^2+^ will be sufficient to trigger the degradation of severed Sarm1-deficient axons. To test this prediction, we transgenically expressed the rat transient receptor potential cation channel subfamily V member 1 (TRPV1) fused to tagRFP in lateralis afferent neurons of Sarm1-mutant zebrafish. Expression of mCherry alone in neurons served as control. TRPV1 is non-selective cation channel that exhibits a high divalent selectivity, and whose activation produces an influx of Ca^2+^ into cells^64^. Rat TRPV1 is activated by temperatures above 43 °C or by the vanilloid capsaicin. Importantly, this TRPV1 is inactive at the temperature used to maintain zebrafish (28 °C), and zebrafish TRPV1 orthologs are insensitive to capsaicin^65^. Therefore, rat TRPV1 expressed in zebrafish offers a tunable tool to elevate axoplasmic Ca^2+^ with excellent temporal resolution. We severed TRPV1-expressing and mCherry-expressing lateralis axons, and 2 h later a vehicle solution or vehicle + capsaicin were added in the water holding the fish (Supplementary Fig. 2a, b). Samples were inspected 90 min later. Severed Sarm1-deficient axons not expressing TRPV1 did not fragment in the presence of capsaicin, or TRPV1-expressing axons bathed in ethanol solution. As hypothesized, we found that Sarm1-deficient TRPV1-expressing axon segments readily degraded in the presence of capsaicin (Supplementary Fig. 2c–f). These results demonstrate for the first time in vivo that elevation of axoplasmic Ca^2+^ downstream of Sarm1 is sufficient to trigger axon degradation.

### Schwann cells are not essential to maintain Sarm1−/− axons

**Fig. 5:**
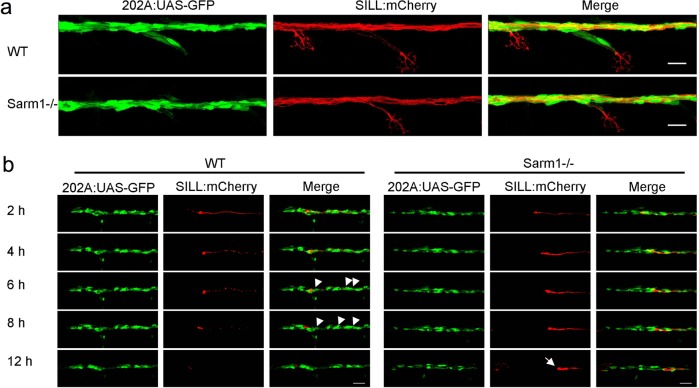
Schwann cells develop normally in Sarm1-deficient zebrafish. **a** Confocal images of a double-transgenic 5dpf larva showing Schwann cells marked by expression of GFP (green) under the control of the Tg[gSAGFF202A] Gal4 driver, and lateralis afferent neurons marked by expression of mCherry under the control of the SILL enhancer (red). Wild type (top), Sarm1 mutants (bottom). Scale bar 20 μm. **b** Images show the indicated time points after axon transection (hours post-injury = hpi) from a videomicroscopic recording of Schwann cells (green) and their interaction with axons (red) in wild type and Sarm1−/−. White arrowheads indicate Schwann cells engulfing axonal debris in the wild type. A white arrow indicates degradation-resistant axon segment in Sarm1−/−. Please note that the proximal axon stump in Sarm1−/− is not visible in these images because it is outside the focal plane.

**Fig. 6:**
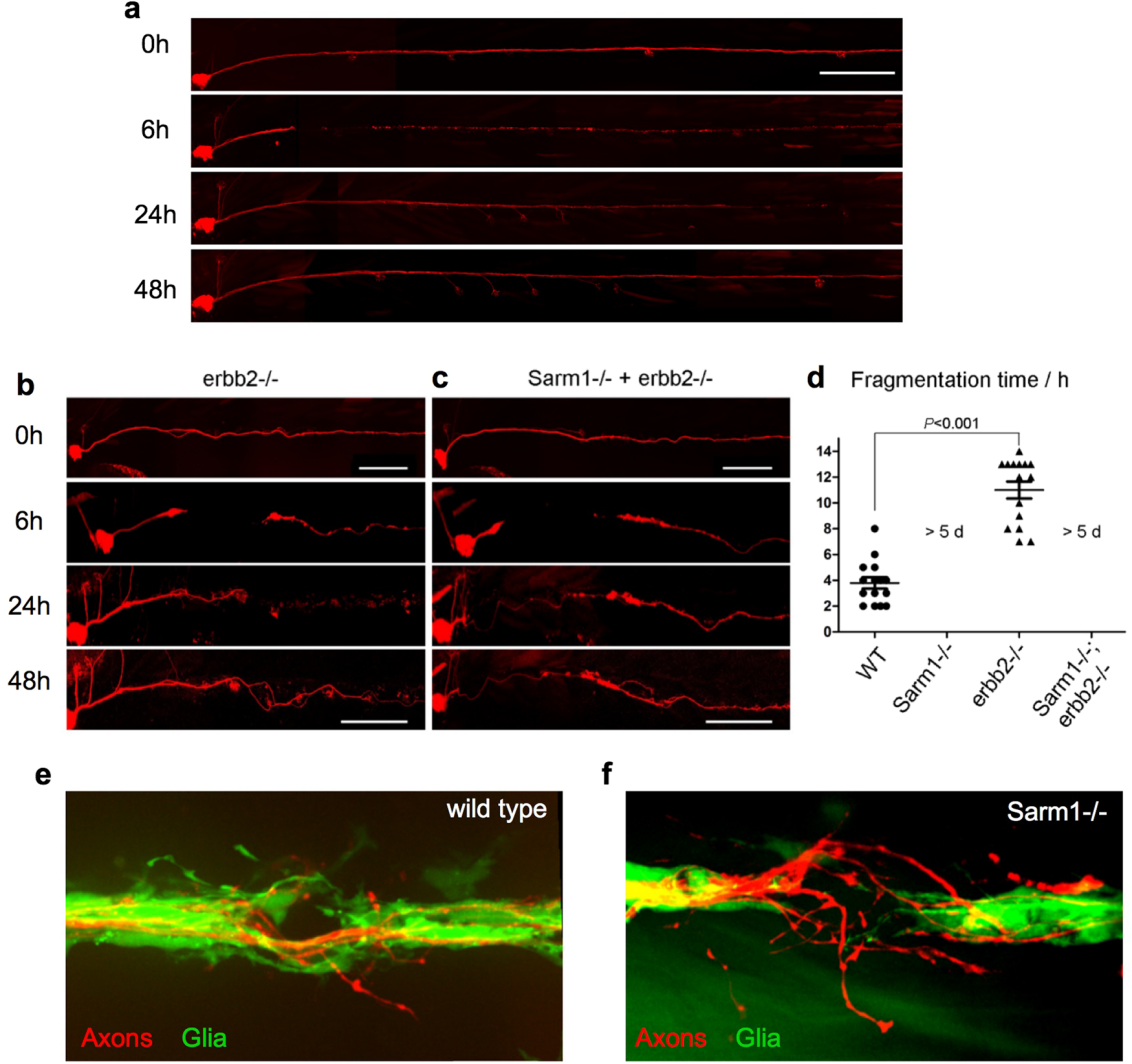
Schwann cells are not essential for the maintenance of Sarm1-deficient sensory axons. **a**–**c** Images of mCherry-expressing (red) transected axons in wild type (**a**), Erbb2−/− mutants (**b**), and Sarm1−/−; ErBb2−/− double mutants (**c**). Scale bar 100 μm. **d** Quantification of transected axon fragmentation in Erbb2−/− and Sarm1−/−; ErBb2−/−. Error bar = SEM, *p* value from one-way ANOVA test, *n* = 15 (each group). **e** Image of from Supplementary Movie 1, showing the discrete local defasciculation of regenerated the sensory fiber (red) and the bridging of the glial gap by Schwann cells (green) in a wild-type specimen. **f** Equivalent experiment, taken from Supplementary Movie 2, showing a more pronounced local defasciculation of the regenerated the sensory fiber (red) in a Sarm1-mutant specimen. Note that the bridging of the glial gap does not occur.

The Schwann cells support, fasciculate, and myelinate sensory axons in vertebrates^66^. We reasoned that delayed axon degeneration by loss of Sarm1 might impact the interaction between axons and Schwann cells. To assess this interaction in vivo, we combined the *sarm1*^*hzm13*^ allele with the triple transgenic line *Tg[gSAGFF202A; UAS-GFP; SILL:mCherry]* to highlight the Schwann cells with green fluorescence and the lateralis afferent neurons with red fluorescence^53,67^. Using high-resolution intravital microscopy, we ascertained that Schwann cells develop normally and fasciculate sensory axons in Sarm1-deficient zebrafish (Fig. 5a). Upon severing, wild-type axons were quickly cleared by the Schwann cells through engulfment of axonal fragments and intracellular degradation of debris (Fig. 5b, Supplementary Movie 4). Thus, we asked if Schwann cells are necessary for the maintenance of severed Sarm1-deficient axons by generating a double mutant zebrafish line concurrently deficient for Erbb2 and Sarm1. In Erbb2-deficient specimens, the distal portion of the severed axons fragmented and were cleared (Fig. 6a–d) but with a significant delay compared to wild-type specimens (Fig. 6a–d). By contrast, in Erbb2/Sarm1 double mutants, severed axons did not fragment or degrade, identically to fish lacking only Sarm1 (Fig. 6c, d). Thus, axon maintenance in Sarm1 mutants occurs independently of the Schwann cells.

When an injury generates a gap in the glia, wound-adjacent Schwann cells actively move and extend cellular projections resembling filopodia to quickly reconstitute a continuous glial scaffold^53^. Although continuous glia are not necessary for the re-growth of the proximal axon stump, it prevents regenerating growth cones from straying, in turn facilitating end-organ de novo innervation and circuit reconstitution^39,41^. Thus, we decided to interrogate Schwann-cell behavior immediately after axon severing and during axon regeneration combining *Tg[gSAGFF202A;UAS-GFP; SILL:mCherry]* double transgene with the Sarm1 mutation. As expected, we found that in wild-type animals, the Schwann cells adjacent to the wound quickly extended filopodia to close the gap in the glial scaffold ahead of axonal regeneration (Supplementary Movie 5). Regrowing fibers then followed these subcellular glial bridges to reconstitute the nerve, suffering mild defasciculation restricted to a small area within the injury (Fig. 6e, Supplementary Fig. 3). In stark contrast, injury-adjacent Schwann cells did not migrate or produce filopodia-like projections in Sarm1 mutants (Supplementary Movie 6). Nonetheless, regenerating axons eventually negotiated the persistent larger gap to grow along the distal glial scaffold. However, the reforming nerves presented more pronounced local defasciculation (Fig. 6f, Supplementary Fig. 4).

### Loss of Sarm1 does not alter Schwann-cell phenotype

**Fig. 7:**
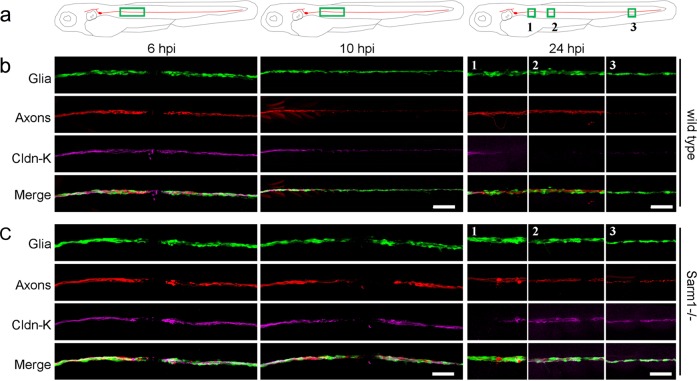
Schwann cells maintain terminal phenotype in Sarm1-deficient zebrafish. **a**–**c** Schematic model of the confocal imaging locations on severed axons (**a**). Confocal images of wild type (**b**) and Sarm1−/− (**c**) specimens in 6, 10, and 24hpi. hpi hour post-injury. The specimens were the Tg[gSAGFF202A;UAS:EGFP; SILL:mCherry] lines and were stained with Claudin-k (magenta) antibody. Scale bar 50 μm.

**Fig. 8:**
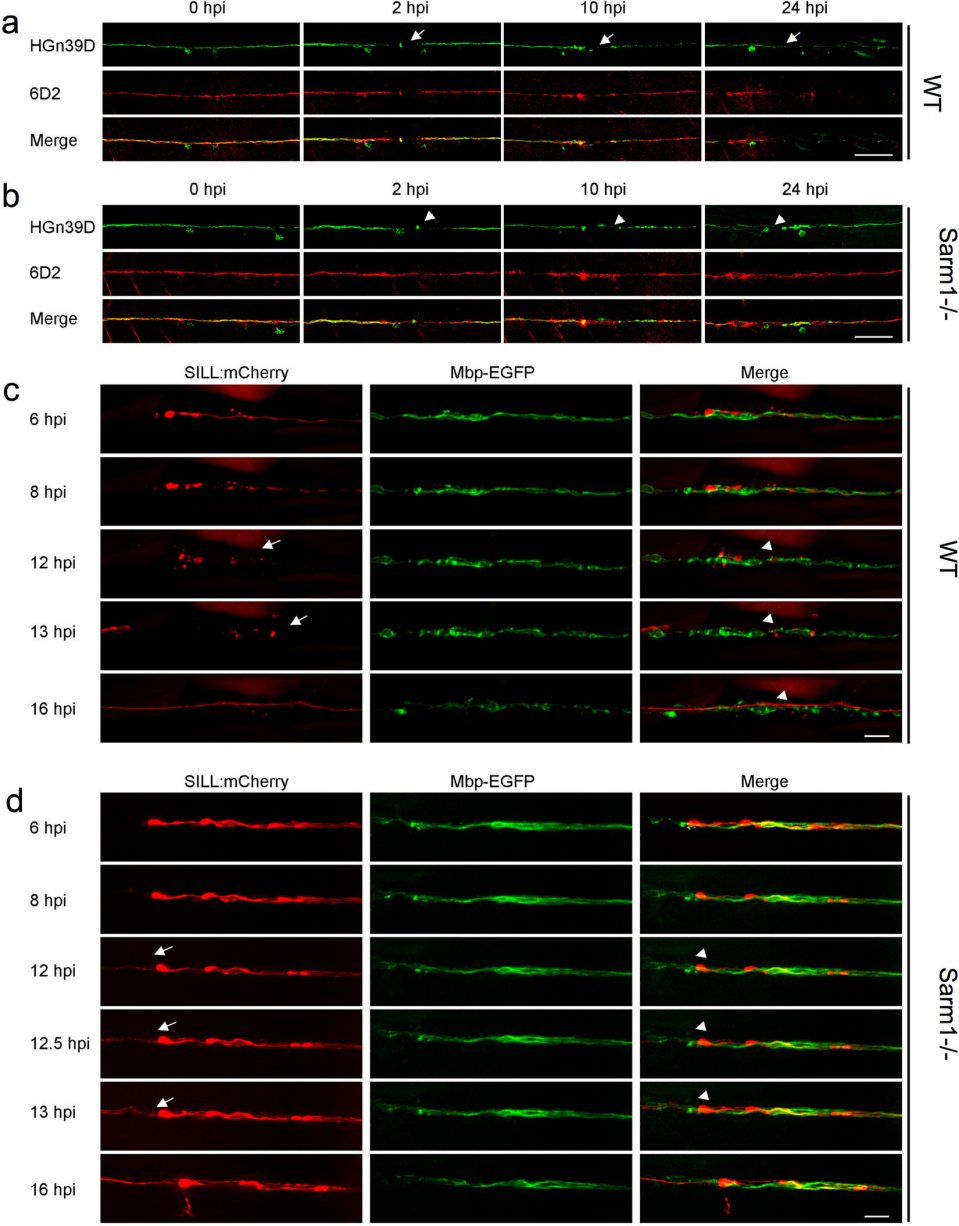
The clearance of the severed axons is not essential for neuronal-circuit repair. **a**, **b** Confocal images of wild type (**a**) and Sarm1−/− (**b**) specimens expressing EGFP in sensory neurons of the lateral line (green) and stained with the monoclonal antibody 6D2. Stainings were performed at indicated time points after axons severing (hpi). The arrows point to the cutting sites. Scale bar is 50 μm. **c** Live imaging of the *Tg[Mbp-EGFP; SILL:mCherry]* after severing. The arrows indicated the fragmented axons and the arrowheads the fragmented myelin. Scale bar 20 μm. **d** Live imaging of the Sarm1−/− in *Tg[Mbp-EGFP; SILL:mCherry]* after severing. Arrows indicate regrowing axons, and arrowheads indicate the juxtaposition between the regrowing axons. Scale bar 20 μm.

Denervated Schwann cells undergo partial dedifferentiation from myelinating to a progenitor-like state as revealed by the loss of expression of terminal-phenotype markers, including myelin and myelin-associated proteins^25,68,69^. The loss of terminal phenotype promotes Schwann-cell proliferation and migration, which enhances their regenerative function^70^. We hypothesized that because Schwann-cell denervation does not occur in Sarm1 mutants, distal Schwann cells may not dedifferentiate. This would explain their lack of phagocytic and protrusive activities after axon transection. Following this rationale, we immunostained samples with an antibody to Claudin-k, which localizes to the junctions between mature Schwann cells and is downregulated in denervated glia^41,71^. In wild-type specimens, Claudin-k remained strongly expressed along the entire length of the lateralis afferent nerve up to 6 h after nerve injury (hpi), suggesting that distal Schwann cells remain mature during distal-axon fragmentation (Fig. 7a, b). Beginning at 10 hpi, however, distal Schwann cells had markedly less Claudin-k than proximal cells. Finally, 24 hpi Claudin-k was conspicuously absent from distal Schwann cells even after axons regeneration had commenced. By contrast, Claudin-k remained strongly expressed after axon severing in Sarm1-mutant animals during the same period, with no apparent difference between Schwann cells located at either side of the wound (Fig. 7a–c). Next, we assessed myelination using the 6D2 monoclonal antibody, which recognizes a carbohydrate epitope in the piscine P0-like myelin glycoproteins IP1 and IP2 (refs. ^72,73^). As with Claudin-k, 6D2 labeling faded in Schwann cells distal to the injury in wild-type specimens (Fig. 8a). Yet, 6D2 labeling remained unchanged in Sarm1 mutants (Fig. 8b). We obtained congruent results when addressing myelination directly in living specimens by using a transgenic line expressing membrane-targeted EGFP under the control of the myelin-binding protein (Mbp:EGFP-CAAX, green) (Fig. 8c, d)^74^. Interestingly, forced degradation of Sarm1-deficient severed axons expressing the rat TRPV1 channel after capsaicin treatment induced associated Schwann cells to dedifferentiate (Supplementary Fig. 2g). Together, these results indicate that Sarm1-deficient sensory axons are maintained independently of Schwann-cell support, and that the clearance of the severed axons is not necessary for regenerating axon growth, pathfinding, myelination, and re-innervation of sensory organs. In addition, they reveal that Schwann cells distal to the injury do not dedifferentiate in Sarm1-mutant specimens.

### Loss of Sarm1 protects Schwann cells from chemical toxins

**Fig. 9:**
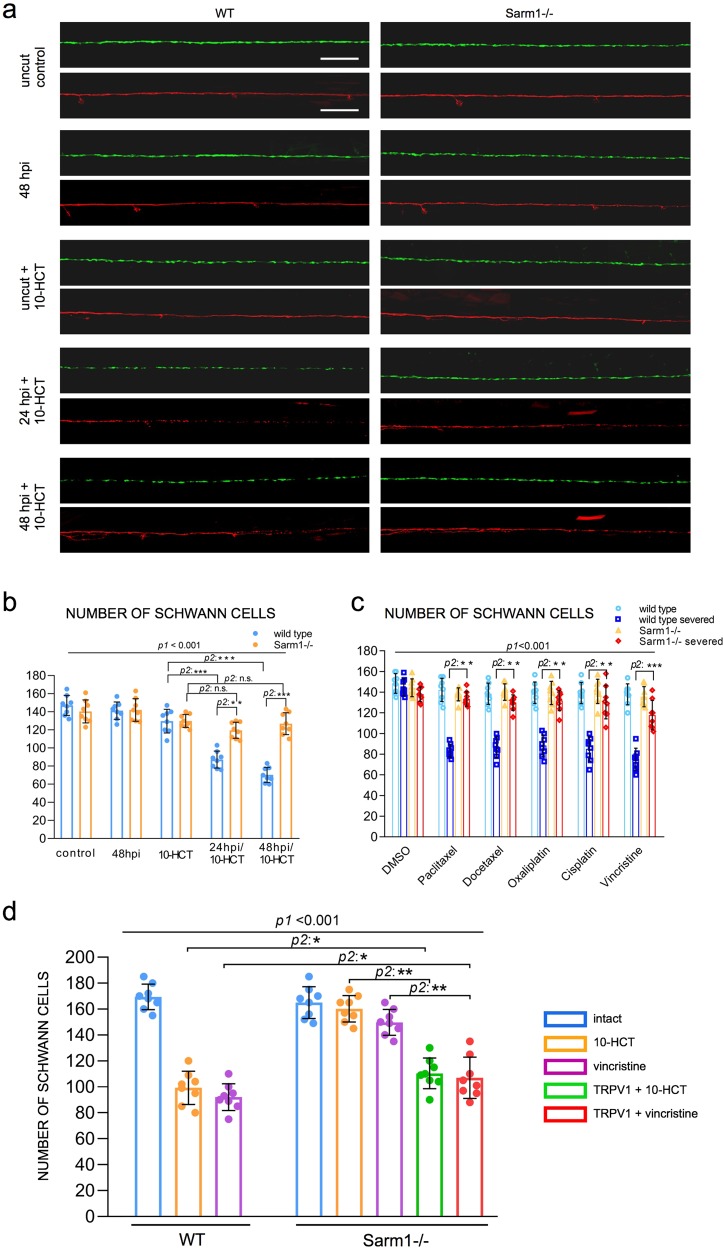
Loss of Sarm1 protects Schwann cells from chemical toxins. **a** Confocal images showing Schwann cells (green) and lateralis sensory axons (red) in a control specimen (in which axons were not transected), in a specimen 48 h after axon transection, and in specimens treated with 10-HCT (10-hydroxycamptothecin). Left column is wild type and right column shows Sarm1−/−. In all cases, the concentration of 10-HCT in water was 40 μm. Scale bar 100 μm. **b** Quantification of the Schwann cells from **a**. Data are shown as mean ± SEM. **p < 0.01, two-way ANOVA, *n* = 8 (each group), followed by *T*-test for two individual group. **c** Quantifi**c**ation of Schwann cells of WT, WT severed, Sarm1−/− and Sarm1−/− severed with the treatment of the indicated chemical compounds for 48 h. Concentrations: Paclitaxel 40 μm, Docetaxel 0.1 μm, Oxaliplatin 500 μm, Cisplatin 50 μm, Vincristine 50 μm. Data are shown as mean ± SEM. *p < 0.05; **p < 0.01, three-way ANOVA, *n* = 8 (each group), followed by *T*-test for two individual group. **d** Quantification of Schwann cells after axon severing, in specimens treated with 10-HCT or Vincristine. The left bar group is wild type. The right bar group is Sarm1−/−, and Sarm1−/− with synthetically eliminated axon segments. Two-way ANOVA, followed by *T*-test for two individual groups.

Many chemotherapeutic agents invariably cause peripheral neurotoxicity, leading to permanent neuronal dysfunction^75–79^. Under conditions of denervation, Schwann cells become strongly susceptible to chemotoxicity^80^. We wondered if the protracted maintenance of severed axons in Sarm1 mutants may suppress glial vulnerability. To test this idea, we treated wild type and Sarm1-mutant zebrafish with several chemical compounds that are under clinical trials or used as first-line treatment for common cancers in humans. First, we used 10-hydroxycamptothecin (10-HCT), which is extremely toxic to denervated glia^81^, and counted Schwann cells using the fluorescence transgenic marker *Tg[gSAGFF202A; UAS:EGFP]*, which is ideal for this experiment because the intensity of green fluorescence does not vary in denervated Schwann cells and, therefore, is independent of the maturity of these glia^41^. We confirmed that 10-HCT does not affect the Schwann cells associated to viable axon (Fig. 9a, b). However, it significantly reduced the number of Schwann cells in 10-HCT-treated wild-type animals after axon severing. By contrast, the number of distal Schwann cells was only marginally affected in Sarm1-mutant specimens (Fig. 9b). Platinum-based, taxanes and some alkaloids are effective chemotherapeutic agents used as standards-of-care for various human malignancies, despite their severe neuropathic effects that include glial destruction^82^. To address their effect on Schwann cell, we treated wild type or Sarm1 mutant zebrafish with cisplatin, oxaliplatin, paclitaxel, docetaxel, and vincristine. We found that upon nerve transection, all these drugs invariably killed injury-distal Schwann cells in wild-type specimens but not in Sarm1 mutants (Fig. 9c). Importantly, none of these drugs affected Schwann cells associated with intact axons, suggesting that axons protect Schwann cells from chemical stress. To confirm this prediction, we forced the degradation of severed axons in Sarm1-deficient zebrafish treated with 10-HCT or vincristine. To this end, we repeated the used of capsaicin to activate rat TRPV1 expressed in a sub-set of lateralis neurons in homozygous mutant fish, in which Sarm1 is absent from every cell, including Schwann cells. This experiment revealed that Schwann cells lacking Sarm1 again become vulnerable to chemotoxicity once severed axons were synthetically eliminated (Fig. 9d), confirming that Sarm1-mediated glioprotection is non-autonomous and depends upon the presence of non-degradable axons.

## Discussion

The sensory neurons that innervate skin, sensory receptors, joints, and muscle communicate peripheral information to the brain, enabling animals to perform the essential activities of daily life. Using zebrafish and a battery of tests that include subcellular structural characterization of sensory neurons and associated Schwann cells, neuronal function, and behavioral assays, we offer a comprehensive and integrated analysis of neuronal and glial response to injury, as well as on the consequences of blocking axon degeneration systemically. It has been well established that the absence of Sarm1 in *Drosophila* and the mouse prevents the degradation of damaged axons^15,83^. Although we were not predicting any differences for the Sarm1 ortholog in zebrafish, assaying functional conservation in our experimental neuronal pathway is not dispensable because some neurotraumatic conditions lead to neuronal loss in the absence of Sarm1. Loss of Sarm1 improves functional recovery after traumatic brain injury in mice, and inhibits vincristine-mediated neurotoxicity^84,85^. Sarm1-deficient mice are viable^19,86^. We found that chronic and systemic loss of Sarm1 is compatible with zebrafish viability and sensorineural function, and demonstrate that the long-term maintenance of non-degradable axon fragments has no detrimental effect on the repair of a sensory circuit. Furthermore, regenerating axons fasciculate and myelinate normally, indicating that they do not compete with non-degradable axons segments for exiting glia. Therefore, we conclude that the axonal destructive and nerve reconstructive processes occur in parallel. In addition, these data suggest that a competitive balance between axon degradation and regeneration does not appear to have shaped the evolution of Sarm1 and, by extension, Wallerian degeneration.

Focusing on Schwann cells, we show that glial cells adjacent to nerve injury in Sarm1 mutants behave dramatically different than those of wild-type specimens. Specifically, Schwann cells in Sarm1 mutants do not migrate or extend projections to bridge the gap in the glial scaffold, indicating that these cells do not directly sense missing intercellular contacts or glial discontinuity. Instead, our findings suggest alternative scenarios. One is that the degradation of axons releases signals that induce Schwann cell to change behavior. In mice, for example, nerve damage promotes mesenchymal behavior of Schwann cells surrounding the wound via TGF-beta signaling, which drives collective Schwann-cell migration across the wound^87^. Although we did not observe Schwann-cell migration, signals derived from injured axons may promote wound-adjacent Schwann cells to extend projections to ^88^ bridge the gap in a similar manner. Interestingly, we observed that filopodia-like structures emerged from Schwann cells at both sides of the injury in wild-type animals, suggesting that repair-inducing signals are likely diffusible, affecting glial cells independently of their association with axons. It remains to be determined if the source of such signals is the damaged axons, the denervated Schwann cells, or other cells in the wound microenvironment.

Upon nerve injury, activated macrophages rapidly accumulate around the wound and contribute to Wallerian degeneration and to axonal regeneration. Specifically, classical pro-inflammatory M1-type phagocytic macrophages remove axonal and myelin debris, whereas anti-inflammatory M2-type macrophages modulate Schwann-cell activity and promote axon regeneration^89^. Immediately after nerve injury, Schwann cells release chemokines that attract or retain macrophages at the wound before axon fragmentation. Importantly, pharmacological activation of macrophage recruitment to the wound microenvironment enhances axon regeneration, whereas decrease of macrophage infiltration inhibits axon regeneration. ROS are conserved early wound signals across species. Injury-mediated ROS production recruits inflammatory cells, including macrophages. Primary sources of ROS after nerve damage are the mitochondria and cellular NADPH oxidases. Studies in ex vivo-cultured neurons indicate that Sarm1 acts downstream of mitochondrial ROS generation. Also, previous studies have characterized Wallerian axon degeneration and regeneration in Erbb2-mutant specimens, but did not address effects on the wound microenvironment and the behavior of macrophages^90,91^. Accordingly, tested in vivo if the loss of Sarm1 as well as the absence of Schwann cells would affect macrophage recruitment, to find that the onset of recruitment, the retention time and number of macrophages at the wound did not differ between wild-type specimens and Sarm1 or Erbb2 mutants. Also, as in wild-type specimens, macrophages in Sarm1 and Erbb2 mutants engulfed debris locally, which were larger within macrophages in Ebbr2 mutants. Thus, the loss of Sarm1 does not affect focal damage resolution by macrophages, which are recruited and activated independently of the Schwann cells. The above findings suggest that activated phagocytic cells other than macrophages and Schwann cells, possibly neutrophils^92^, engulf and clear distal-axon fragments after damage, and that this process does not occur in Sarm1-deficient animals.

In conclusion, current mechanistic details about Sarm1 function derived from independent observations that had been extrapolated from a wide variety of experimental systems. This has made it difficult to synthesize findings and reconcile some conflicting observations. Here, we have exploited a powerful in vivo genetic system to comprehensively study the consequence of systemic loss of Sarm1 from the subcellular to the organismal level in a single vertebrate system. By generating zebrafish carrying loss-of-function mutations in Sarm1, we confirmed and deepen previous findings from *Drosophila*, the mouse, and cultured cells. Several novel insights derive from the work presented here. First, that loss of Sarm1 is well tolerated by the animal. Second, that neuronal-circuit repair is not contingent upon rapid clearance of damaged axons. Third, that Schwann cells are not necessary for maintenance of severed axons in vivo. Fourth, that after the axotomy Schwann cells distal to the cut site do not dedifferentiate in Sarm1 mutants in vivo. Fifth, that the protracted maintenance of transected axons dramatically improves Schwann-cell tolerance to chemotoxicity after nerve injury after neurotrauma. This scenario is likely to occur in the clinical setting because nerve trauma is an inescapable consequence of surgical interventions, chronic metabolic dysfunction including diabetes, and pharmacological treatments such as antibiotics and anæsthesia that increase cellular stress^82^. Therefore, these findings are of obvious pathophysiological significance. Crucially, these data lend strong support to the idea that direct interventions to systemically inhibit axon degradation are promising strategies to reduce chronic consequences of neurotrauma. Because TIR domain dimerization is necessary and sufficient to degrade NAD+, it renders Sarm1 amenable to inhibition by small molecules, as it has been demonstrated for the TIR domain of TLR2 (ref. ^93^). Our findings encourage the development of Sarm1 inhibitors for therapeutic applications^18,29^.

## Methods

### Zebrafish strains and husbandry

Zebrafish (*D. rerio*) were maintained in a centralized facility in accordance to guidelines by the Ethical Committee of Animal Experimentation of the Helmholtz Zentrum München, the German Animal Welfare act Tierschutzgesetz §11, Abs. 1, Nr. 1, Haltungserlaubnis, to European Union animal welfare, and to protocols number Gz.:55.2-1-54-2532-202-2014 and Gz.:55.2-2532.Vet_02-17-187 from the “Regierung von Oberbayern”, Germany. The transgenic lines Tg[UAS:EGFP], Tg[HGn39D] and Tg[SILL:mCherry]^67^, Tg[gSAGFF202A]^41^, Tg[UAS:GCaMP7a]^94^, Tg[mbpa:tgRFP-CAAX]^tum102Tg^ (also known as Tg[MBP:tgRFP])^95^ and Erbb2 mutants^41,59^ have been previously published. The Tg[Sarm1−/−] was generated by CRISPR/Cas9-mediated mutagenesis.

### Sarm1 mutagenesis

We used CRISPR/Cas9-mediated genome modification to generate mutations in exon 1 of Sarm1. Cas9 mRNA and sgRNAs were co-injected into one-cell stage embryos. Cas9 mRNA was generated in vitro from *Pme*I-linearized CAS9 vector (pMLM3613) using the Ambion^TM^ mMESSAGE mMACHINE T7 Kit. The mRNA was purified with the RNA easy kit (Qiagen). To generate the sgRNAs, the target exon was sequenced and the sequence information was used to design oligonucleotides for the sgRNA guide vector (pDR274) using the on-line tool “ZiFiT Targeter software package” (http://zifit.partners.org/*)*^96^. The sgRNA sequence for exon 1 of Sarm1 is 5′-GGGACTTGGAAGAGACCCGC-3′. The annealed oligonucleotide was cloned into the *Bsa*I-digested pDR274 vector using T4 ligase (NEB M0202). Resulting clones were sequenced to verify correctness, and then linearized with *Dra*I. The purified linearized DNA fragment was employed to generate the sgRNA using the T7 MEGAscript kit (Ambion^TM^). By out-crossing adult fish resulting from injection, we obtained germ-line transmission of two independent alleles: *sarm1*^*hzm13*^ and *sarm1*^*hzm14*^. For genotyping mutant carriers, we used the primers: Forward: 5′-GATTTGCCGTTATCTCTCCA-3′, Reverse: 5′-TCAAGCAGTTTGGCAGACTC-3′.

### DNA constructs

The DNA constructs SILL:mCherry, SILL:Gal4 (ref. ^67^), UAS:Synapsin1-GFP^53^, and mbp:EGFP-CAAX have been previously described. The vectors pMLM3613 (42251) and pDR272 (42250) were purchased from Addgene. The plasmids UAS:TRPV1-tagRFP, the coding sequence of rat TRPV1 containing the E600K mutation fused to tagRFP, was synthesized by Genecat and the expression construct was generated using Tol2 kit. The constructs SILL:mito-mCherry, SILL:Sarm1-v2a-mCherry, SILL:Kaede, SILL:mitoRGECO, and SILL:erGCaMP3 were generated using Tol2 kit. Mitochondria in lateralis neurons were marked by expressing the mitochondria-targeting sequence from the cytochrome-*C* oxidase subunit 8A fused to mCherry. The plasmids containing mito-RGECO and er-GCaMP3 (ref. ^97^) were a gift of D.W. Raible (University of Washington).

### Antibodies and immunostaining

Whole-mount immunostaining was performed as following. First, samples of zebrafish embryos or larvae were fixed by immersion in ice-cold 4% paraformaldehyde diluted into phosphate-buffered saline buffer containing 0.2% Tween-20 (PBST), and incubated overnight at 4 °C. Then, the samples were washed at room temperature (RT) with PBST three times, 10 min per wash, and subsequently blocked in 10% bovine serum albumin (BSA), also at RT for 1 h. Next, the samples were incubated in primary antibodies at 4 °C overnight. Next, the samples were washed in PBST for 2 h, changing to fresh buffer every 30 min. Finally, they were incubated with secondary antibodies at 4 °C overnight. Primary antibodies and concentrations: mouse anti-Acetylated tubulin, 1:1000 (Sigma T7251); rat anti-Claudin-k, 1:500 (gift from T. Becker, University of Edinburgh, UK)^71^; mouse 6D2, 1:5 (gift from Dr. G. Jeserich, University of Osnabrück, Germany)^73^. Secondary antibodies used were at the following concentrations: donkey anti-Mouse Alexa Fluor® 555, 1:200, Abcam ab150106; donkey anti-Rat IgG H&L (Alexa Fluor® 647) pre-adsorbed, 1:200, Abcam ab150155). Samples were washed in PBST for 30 min and mounted in Vectashield 1 day before microscopic examination. Imaging of fixed samples was done with a laser-scanning confocal microscope (LSM 510, Carl Zeiss).

### Intravital microscopy

For videomicroscopy, larvae were anesthetized with MS-222 (0.013% M/V) in Danieau’s and mounted in 0.8% low melting-point agarose on 35 mm glass-bottom Petri dishes. Samples were gently pressed against the glass using a hair-loop glued to the tip of a glass pipette, as previously described. The agarose dome was immersed in Danieau’s with MS-222. Images of cells were acquired using a spinning-disc microscope with a ×40 air objective at 28.5 °C^58^. Z-stacks were set to 0.8–1.2 µm intervals. Time intervals were 10 min or 15 min per stack. The resulting raw data were processed, assembled, and analyzed with ImageJ.

### Western blot assay

Wild type and mutant larvae were anesthetized and killed 5 days-post-fertilization. Samples were homogenized in ice-cold RIPA buffer (with protease inhibitor cocktail from Roche (Cat.04693159001). After homogenization, the samples were incubated on ice for 30 min for further lysis. The resulting lysate was centrifuged at 1200 r.p.m. at 4 °C, and the supernatant was taken for the BCA assay. The supernatant was diluted in loading buffer and boiled at 99 °C for 5 min. Next, the samples were run in SDS-PAGE and transferred onto a PVDF membrane. After blocking the membrane in 5% skimmed milk (diluted in PBST) for 1 h, the membrane was incubated with the primary antibody (rabbit anti-Sarm1, 1:500, ANASPEC 55381; Mouse anti-β-Tubulin, 1:2000, Sigma T5168) at 4 °C overnight. The next day, the membrane was washed and incubated with HRP-labeled secondary antibody (Peroxidase-Affini Pure Goat Anti-Mouse IgG (H + L), 1:10000, Jackson Immuno Research 115035003; Peroxidase-Affini Pure Goat Anti-Rabbit IgG (H + L), 1:10000, Jackson ImmunoResearch 115035144) for 1 h. Images were acquired by developing the membrane with ECL (Pierce™ ECL Western Blotting Substrate, Thermo Fisher, 32109).

### Laser microsurgery

To mark lateralis sensory neurons individually, DNA of the SILL:mCherry construct was injected into eggs of Tg[HGn39D], Tg[HGn39D; Sarm1−/−], Tg[SILL:mCherry; gSAGFF202A; UAS:EGFP] or Tg[SILL:mCherry; gSAGFF202A; UAS:EGFP; Sarm1−/−] zebrafish. Resulting larvae were selected according to red fluorescence in lateralis neurons. Selected samples were mounted into agarose as described above, and their peripheral axons were targeted an ultraviolet laser (350 nm) using the iLasPulse system (Roper Scientific AS, Evry, France), as described previously. The laser beam was delivered using a ×63 water-immersion objective^50,53,58^. The laser pulses were calibrated and applied to the target area until a clear gap in the axons was visible. The samples were observed again 1 hpi to confirm complete axon transection.

### Quantification of mitochondrial density and motility

To analyze mitochondria in sensory axons, we generated kymographs of mito-mCherry fluorescent spots using the Multi-Kymograph tool of the Fiji software (http://fiji.sc). The movement of mitochondria was determined by the slope of the lines drawn over time, and the direction of movement was determined by the moving mitochondria crossing the time line (vertical) in the kymographs. The data were analyzed with Python scripts and the Graphpad Prism software.

### Calcium imaging

For calcium imaging in lateralis neurons, Tg[SILL:Gal4; UAS:GCaMP7a] double-transgenic larvae were anesthetized and mounted in 0.8% low melting-point agarose on a 35 mm glass-bottom Petri dish. Imaging was acquired through a ×63 water-immersion objective with an exposure time of 400 ms. Laser-mediated axon transection was done after the fourth imaging of the time series. Next, live videomicroscopy was done for 2 min at a frame rate of 400 ms at 28.5 °C. The raw data were analyzed with ImageJ. To quantify the calcium signal, the images were processed to ImageJ. The region of interest (ROI) was selected and measured the value with time point. GCaMP or RGECO intensity changes were calculated as follows: ∆*F*/*F*0 = (*F*–*F*0)/*F*0, where *F*0 is the value of the fluorescent signal before axons were transected, and *F* is the value of the fluorescent signal with time point after axon severing^97^.

### Chemogenetics

For chemogenetic experiments, we co-injected the SILL:Gal4 with UAS:mCherry or UAS:ratTRPV1-tagRFP into the Tg[UAS:GCaMP7a] or Tg[HGn39D; Sarm1−/−]. The positive larva with SILL:Gal4; UAS:ratTRPV1-tagRFP; UAS:GCaMP7a expression was used to activate TRPV1 channels in zebrafish by incubation in 5 μM capsaicin (Sigma, M2028) and subsequent live imaging for 1 h of the mounted and anesthetized embryo. Images were acquired through a ×63 water-immersion objective with an exposure time of 400 ms. For experiments with the Sarm1 mutant larvae, the HGn39D with SILL:mCherry or ratTRPV1-tagRFP positive animals were laser axotomized. The larvae were treated with 10 μM capsaicin or ethanol (1:1000, v/v) 2 h after transection. At 1.5 h after capsaicin treatment, images were taken by spinning-disc microscopy.

### Behavioral assays

For the touch-mediated escape response, 2 dpf embryos were gently dechorionated and kept in Danieau’s solution at 28 °C for at least 1 h. Embryos were place into a flat uncovered Petri dish containing Danieau’s and were recorded with a high-speed camera (NX4 series, Imaging solution, GmbH). Video recording was launched and a randomly chosen embryo was touched with a blunt glass needle until it evoked a reaction. Recordings were done under white-light illumination over 150 s at a rate of 200 frames per second (fps). The swimming trajectories were obtained with 3D Particle Tracker plugin, ImageJ software. The further quantification and statistics were using Python.

### Statistics and reproducibility

The Student’s *t*-test (two tailed), ANOVA test, *T*-test (Tukey test), and Wilcoxon rank sum test were applied using Python scripts. Error bars in all figures are standard errors of the mean (SEM).

### Reporting summary

Further information on research design is available in the Nature Research Reporting Summary linked to this article.

## Supplementary information

Supplementary Movie 1

Supplementary Movie 2

Supplementary Movie 3

Supplementary Movie 4

Supplementary Movie 5

Supplementary Movie 6

Description of Additional Supplementary Files

Supplementary Information

Reporting Summary

## Data Availability

All data supporting the findings of this study are available within the paper and its supplementary information. Reagents generated during the study are available from the corresponding author on reasonable request.
