# Supplementary material for: Systemic loss of Sarm1 protects Schwann cells from chemotoxicity by delaying axon degeneration
Source: Commun Biol. 2020 Jan 30;3:49. doi: 10.1038/s42003-020-0776-9 (PMC6992705; doi:10.1038/s42003-020-0776-9)
Supplement: Supplementary file 7 — Description of Additional Supplementary Files [file 42003_2020_776_MOESM7_ESM.doc]

**Descriptions of additional supplementary files**

**Supplemental Movie 1.** Videomicroscopic imaging of a double transgenic *Tg[SILL:mCherry; Mfap4-memEGFP]* 4dpf wild-type specimen in which the  mCherry(+) axons were severed by a laser pulse. The video represents for 13 hours  of continuous imaging at a 5-minute temporal resolution. Axons (red) and macrophages (green) were visualized relative to the cut (proximal is on the left and distal in on the right).

**Supplemental Movie 2.** Videomicroscopic imaging of a double transgenic

*Tg[SILL:mCherry; Mfap4-memEGFP]* 4dpf specimen that was mutant for

Sarm1, in which the mCherry(+) axons were severed by a laser pulse. The video represents for 13 hours of continuous imaging at a 5-minute temporal resolution. Axons (red) and macrophages (green) were visualized relative to the cut (proximal is on the left and distal in on the right).

**Supplemental Movie 3.** Videomicroscopic imaging of a double transgenic

*Tg[SILL:mCherry; Mfap4-memEGFP]* 4dpf specimen that was mutant for

Erbb2, in which the mCherry(+) axons were severed by a laser pulse. The video represents for 13 hours of continuous imaging at a 5-minute temporal resolution. Axons (red) and macrophages (green) were visualized relative to the cut (proximal is on the left and distal in on the right).

**Supplemental Movie 4.** Videomicroscopic imaging of a 4dpf wild-type specimen expressing GFP in Schwann cells, and mCherry in lateralis sensory neurons, showing the engulfment of axonal fragments by these glia after axon severing, as well as axonal regrowth after clearance of severed axons. It was recorded during 12 hours at 5-minute intervals.

**Supplemental Movie 5.** Videomicroscopic imaging of a 4dpf wild-type specimen carrying three transgenes Tg[SILL:mCherry; gSAGFF202A; UAS:EGFP], showing the dynamics of axons and Schwann cells after axonal transection. Sensory axons are shown in red and Schwann cells in green. The movie is a maximal projection of confocal stacks. It was recorded during 12 hours at 10-minute intervals. The top panel shows the merged images, the middle panel the Schwann cells, while the bottom panel the axons.

**Supplemental Movie 6.** Identical experiment as shown in Supplemental Movie 1, but conducted in a Sarm1-mutant specimen. It was recorded during 12 hours at 10-minute intervals. The top panel shows the merged images (SILL:mCherry; gSAGFF202A; UAS:EGFP), the middle panel the Schwann cells (gSAGFF202A; UAS:EGFP), and the bottom panel the axons (SILL:mCherry).
